# Centennial scale sequences of environmental deterioration preceded the end-Permian mass extinction

**DOI:** 10.1038/s41467-023-37717-0

**Published:** 2023-04-14

**Authors:** Ryosuke Saito, Lars Wörmer, Heidi Taubner, Kunio Kaiho, Satoshi Takahashi, Li Tian, Masayuki Ikeda, Roger E. Summons, Kai-Uwe Hinrichs

**Affiliations:** 1 MARUM - Center for Marine Environmental Sciences & Faculty of Geosciences, University of Bremen, 28359 Bremen, Germany; 2 Department of Earth, Atmospheric and Planetary Sciences, Massachusetts Institute of Technology, 45 Carleton Street, Cambridge, MA 02142 USA; 3 Graduate School of Sciences and Technology for Innovation, Yamaguchi University, 1677-1 Yoshida, Yamaguchi City, 753-8512 Japan; 4 Japan Science and Technology Agency, PRESTO, 4-1-8 Honcho, Kawaguchi, Saitama, 332-0012 Japan; 5 Department of Earth Science, Tohoku University, Sendai, 980-8578 Japan; 6 Department of Earth and Environmental Sciences, Graduate School of Environmental Studies, Nagoya University, Nagoya, 464-8601 Japan; 7 The Key Laboratory of Biogeology and Environmental Geology and Faculty of Earth Science, China University of Geosciences Wuhan, Wuhan, 430074 China; 8 Department of Earth and Planetary Science, University of Tokyo, Bunkyo, 113-0033 Japan

**Keywords:** Carbon cycle, Geochemistry, Palaeoclimate

## Abstract

The exact drivers for the end-Permian mass extinction (EPME) remain controversial. Here we focus on a ~10,000 yr record from the marine type section at Meishan, China, preceding and covering the onset of the EPME. Analyses of polyaromatic hydrocarbons at sampling intervals representing 1.5–6.3 yr reveal recurrent pulses of wildfires in the terrestrial realm. Massive input pulses of soil-derived organic matter and clastic materials into the oceans are indicated by patterns of C_2_-dibenzofuran, C_30_ hopane and aluminum. Importantly, in the ~2,000 years preceding the main phase of the EPME, we observe a clearly defined sequence of wildfires, soil weathering, and euxinia provoked by the fertilization of the marine environment with soil-derived nutrients. Euxinia is indicated by sulfur and iron concentrations. Our study suggests that, in South China, centennial scale processes led to a collapse of the terrestrial ecosystem ~300 yr (120–480 yr; ± 2 s.d.) before the onset of the EPME and that this collapse induced euxinic conditions in the ocean, ultimately resulting in the demise of marine ecosystems.

## Abstract (web summary)

The exact drivers for the end-Permian mass extinction remain controversial. This study reveals a turning point with the exhaustion of the terrestrial input and a strong fertilization of the marine realm leading to the demise of marine ecosystems.

## Introduction

The end-Permian mass extinction (EPME) was the most severe of the Phanerozoic, impacting both the marine and terrestrial biospheres with ~90% marine species loss and ~70% land-based vertebrate family loss^1^. The favored hypothesis is that contemporaneous emplacement of the Siberian Traps Large Igneous Province (LIP) was the primary driver^2,3^. The Siberian Traps eruptions could have induced a chain of deleterious environmental impacts, including shallow marine anoxia^4^, lethal temperatures^5^, emission of toxic metals^6^, emission of climate-modifying volcanic gases (e.g., SO_2_ and CO_2_), wildfires, destruction of atmospheric ozone, acid rain, and the promotion of weathering of fresh volcanic rocks^1^.

As Siberian Traps eruptions have a reported duration of at least 900 kyr^3,7^, it remains to be explained what triggered the 60 ± 48 kyr marine mass extinction event^8,9^ within the Permian-Triassic transitional interval^10,11^. Heterogeneity of Siberian Traps activity concerning the magnitude of eruptions and emplacement style (e.g., intrusive vs. eruptive) has been introduced as a potential explanation for high-intensity events at decadal to centennial time scales^3,10,11^. Given the difficulty in elucidating a decadal- to centennial-scale volcanic history from the volcanic rock relics in Siberia, various direct or indirect chemical tracers (e.g., Hg) have been introduced to track LIP activity^12–15^. Some of these studies suggest that the strong volcanic activity at the end-Permian was pulsatile^16^, and that for both marine and terrestrial extinctions to occur, the destruction of terrestrial ecosystems had to take place first^17–19^. Global climate simulations of flood basalt gas emissions, described by sulfur chemistry and aerosol microphysics coupled with atmosphere-ocean circulation, predict perturbations on scales of several hundred years^11^. Detailed, high-resolution (mm-scale or higher) investigations of tracers for volcanic activity, terrestrial ecosystem collapse, and marine environmental degradation are thus needed to clarify the sequence of these events and their causal relation to the biotic extinction.

Among the suite of sedimentary molecular biomarkers, polyaromatic hydrocarbons (PAHs) have been proposed to track combustion events such as wildfires, volcanic activity, and asteroid impacts^20–22^. Additionally, C_2_-dibenzofuran (C_2_-DBF), a compound of predominantly terrestrial origin, has been detected in sediments^17^. Furans are produced by the dehydration of polysaccharides such as cellulose, which are typically abundant in litter and soils^23^. Thus, PAHs and C_2_-DBF have previously been used, at cm-resolution or coarser, as a marker of the devastation of terrestrial ecosystems at the EPME^17,21,24^.

**Fig. 1:**
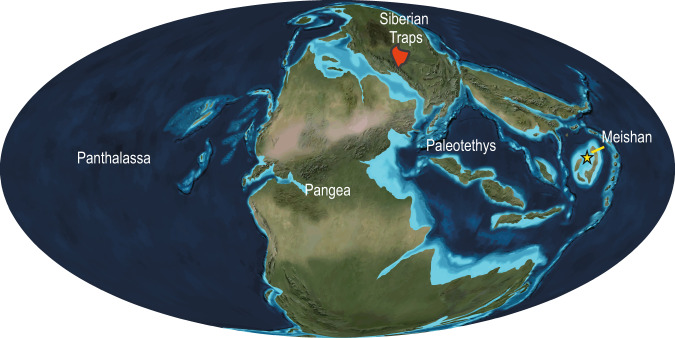
Late Permian global paleogeography. The Meishan section and Siberian Traps are indicated by yellow star and red area, respectively. source map © 2020 Colorado Plateau Geosystems Inc.

The method of mass spectrometry imaging (MSI) was recently implemented for geoscientific applications to extract climate records from marine sediments at ultra-high temporal resolution^25,26^. We here conduct an MSI analysis of molecular biomarkers at 0.1 mm resolution on the uppermost Permian of the Changhsing Formation in the marine Meishan section, i.e., the Global Stratotype Section and Point (GSSP) for the Permian-Triassic boundary^27^. During the late Permian, the Meishan region in South China was located in the low-latitude eastern Paleotethys Ocean (Fig. 1). According to conodont age-diagnostic fossil zones, the Meishan section covers the upper Permian to Lower Triassic, including the EPME^28,29^, archiving data of terrestrial and marine origin. We pair MSI analysis with records of elemental composition at 0.05-mm resolution obtained by micro-X-ray fluorescence spectroscopy (micro-XRF) to provide insight into the contribution of clastic material and provenance as well as redox conditions^30,31^. Moreover, in order to validate the robustness of MSI analysis, conventional molecular biomarker analysis by gas chromatography-mass spectrometry (GC-MS) is conducted at 1–5 cm-resolution. Our combined biomarker and elemental data thus reveal the fine-scale pattern of environmental change in the terrestrial and oceanic realms leading to the EPME recorded in the near-shore marine succession at Meishan.

## Results and discussion

### Fire events on a multi-century scale

**Fig. 2:**
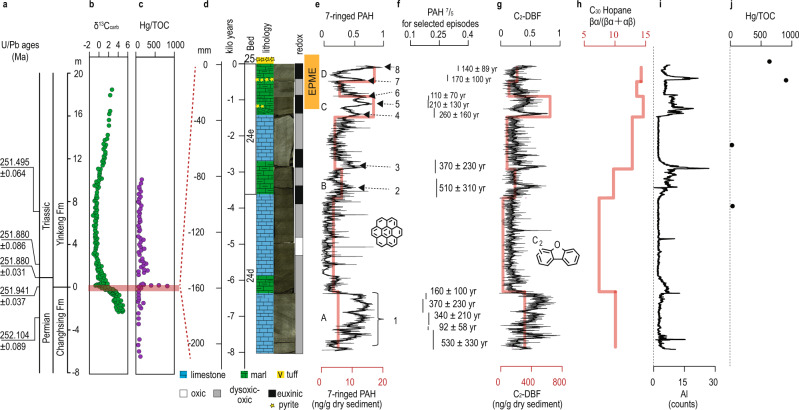
Abrupt environmental deterioration leading to the end-Permian mass extinction: chemostratigraphy of molecular biomarkers and aluminum abundances from the Meishan section, South China. **a**–**c** A stratigraphic record (−8 to 20 m) of U/Pb ages^8^ (**a**), stable carbon isotopic composition of carbonate^18^ (**b**) as a gauge of the carbon cycle and Hg/TOC ratio^18^ (**c**) representing volcanogenic Hg concentrated in terrestrial ecosystems. **d**–**j** An expanded stratigraphic record of the top 200 mm below the base of Bed 25 (0 m) for the lithological column with ages and redox conditions based on framboidal pyrite data^39^ (**d**), 7-ringed PAH (**e**) as a combustion marker, PAH^7^/_5_ (**f**) as a combustion temperature marker, C_2_-DBF (**g**) and C_30_ hopane βα/(βα + αβ) (**h**) as markers for soil input, aluminum (relative abundance) (**i**) as a marker for clastic input, and the Hg/TOC ratio (**j**). In **d**, the linear high-resolution age model was constructed using a mean-weighted sedimentation rate (2.6 cm/kyr)^8^ (see text). EPME is after ref. ^21^. In **e**, letters A–D identify intervals of increased PAH concentrations based on the GC analysis, while numbers 1–8 identify single events of increased PAH input based on the MSI analysis. In **f**, solid vertical lines indicate mean values of PAH^7^/_5_ for each of the eight events, while numbers along the lines indicate the inferred duration of each event (mean ± 2 s.d.). In **e**, **g**, **h**, the vertical red lines on each plot are the corresponding depth range of GC-MS data for each sample, black lines show MSI-based data with 0.1 mm resolution. In **e** and **g**, seven-ringed PAH and C_2_-DBF for MSI-based data are normalized to their maximum values; solid black lines are derived from locally weighted scatterplot smoothing (lowess) of the data.

Strong variability in the intensity of combustion events is identified in the ~10,000 yr succession leading to the EPME^32^, based on the PAH data. Four millennial-scale intervals of increased PAH accumulation are identified through GC- and MSI-based analyses (intervals A–D in Fig. 2 and Supplementary Fig. 1). The agreement between both techniques confirms the robustness of the obtained data. A sub-decadal resolution offered by MSI further reveals the pulsed nature of the PAH signal. Eight major combustion episodes are clearly distinguishable (events 1–8 in Fig. 2 and Supplementary Fig. 2) coupled with numerous smaller peaks. We estimate the mean duration of these events to be 270 ± 150 yr (mean ± s.d.) (Supplementary Table 1). Their intensity and frequency increased towards the top of the analyzed section, an observation that is also supported by wavelet analysis (Supplementary Fig. 3).

**Fig. 3:**
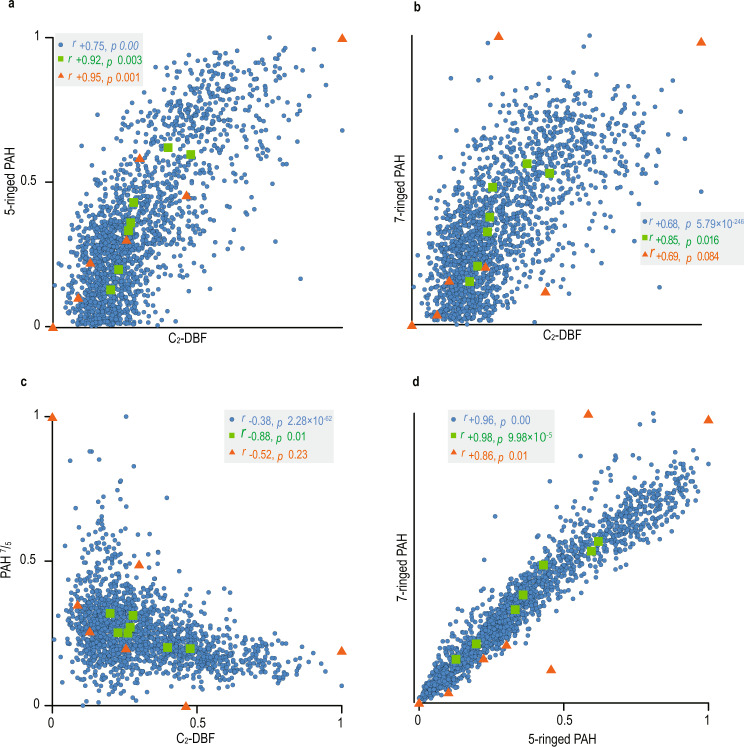
Pyrogenic PAHs vs. soil organic matter. Cross-plots of molecular biomarkers for pairs of five-ringed PAH and C_2_-DBF (**a**), seven-ringed PAH and C_2_-DBF (**b**), PAH^7^/_5_ and C_2_-DBF (**c**), and seven- and five-ringed PAHs (**d**). The seven- and five-ringed PAHs and PAH^7^/_5_ are shown as markers for combustion and combustion temperature, respectively (see text), and C_2_-DBF as markers for soil organic matter. Blue circles: MSI data; orange triangles: GC-MS data; green squares: the mean values of MSI data corresponding to the stratigraphic range for each sample of GC-MS data. Pearson’s *r* values (*r*) and *p* values (*p*) are shown in the plots. For MSI data, the sample size is 1863, 1821, 1776, and 1823 in plots a–d, respectively. For MSI mean and GC-MS data sample size is *n* = 7 in all plots. MSI and GC-MS data are normalized to their maximum values. Because of the large sample size of the MSI data, its *p* value is prone to be smaller than <0.05, so the correlation coefficient and *p* value were also calculated for the mean value of MSI data. Note that as the contribution of C_2_-DBF increases, the PAH^7^/_5_ ratio decreases.

Records of C_2_-DBF and PAHs are positively correlated (Figs. 2, 3). We interpret this as the result of wildfires that removed the vegetative cover (See Supplementary Discussion), thus leaving soils exposed to enhanced weathering and erosion, as observed in modern environments and discussed for the EPME^33^. Simultaneously, this correlation suggests that PAHs were mainly transported to the marine environment through surface runoff, along with abundant soil-derived organic matter. From interval B upwards, elevated aluminum abundances coincide with peaks of PAHs. Accumulation of Al in the marine record could be indicative of weathering, and/or Al leaching from soils due to acid rain^1,34^.

The ratio between different PAH species can provide insight into the formation conditions of the PAHs detected in sedimentary rocks. Larger molecules of PAHs (e.g., the seven-ringed coronene) require a higher temperature to form due to kinetic limitations to the successive addition of rings^35^. Consequently, the geological record of past volcanogenic combustion^20^ and asteroid impacts^22^ show a higher relative abundance of larger ringed PAHs, especially coronene. At the end-Permian, injections to the stratosphere from Siberian Traps volcanism resulted in a widespread distribution of relatively enhanced coronene concentrations and a positive correlation with mercury (Hg)^21^. By contrast, in modern wildfire PAH records, seven-ringed PAH are less abundant due to the relatively low combustion temperature^36^. We use the ratio of seven-ringed vs five-ringed PAHs (PAH^7^/_5_)^20^ as a proxy for investigating the intensity of combustion events in the GSSP Meishan record.

**Fig. 4:**
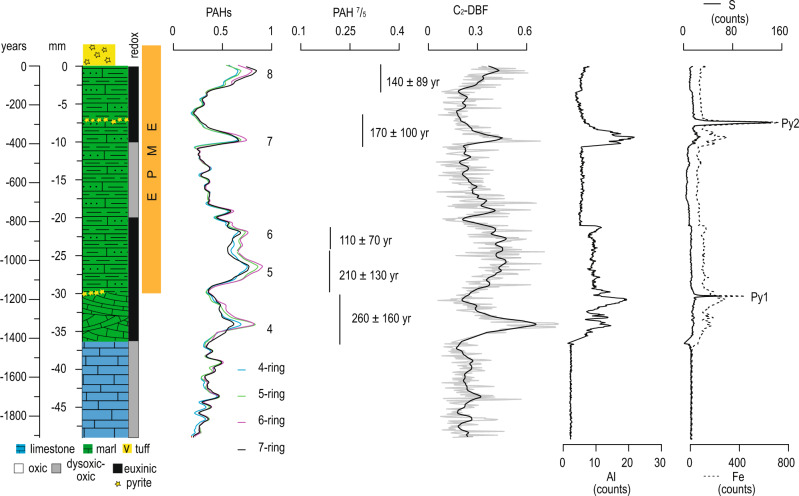
A high-resolution record from the top of Bed 24e. The four- to seven-ringed PAHs and mean values of PAH^7^/_5_ for events 4–8 are shown as combustion markers and combustion temperature, respectively. C_2_-DBF and Al are shown as markers for soil and clastic input, respectively. S and Fe are shown to indicate the existence of pyrite spikes. The numbers from 4 to 8 along the plot are denoted as episodes of increased PAH input. The redox bar is after the framboidal pyrite data of ref. ^39^. Py 1 and Py 2 are indicating pyrite spikes. EPME is after ref. ^21^. The relative age model is based on a mean-weighted sedimentation rate (2.6 cm/kyr)^8^ (see text).

The PAH^7^/_5_ values remain relatively low across the entire sampled record, with the exception of the ~3000 yr period between intervals A and B, where the lowest concentrations of PAH and C_2_-DBF, but the highest values of PAH^7^/_5_, are observed (Supplementary Fig. 1). The association of low PAH^7^/_5_ values with highest values of the soil marker C_2_-DBF suggests that soil-derived PAHs are dominated by compounds with smaller numbers of rings (Fig. 3). Accordingly, the high values of PAH^7^/_5_ between intervals A and B (Supplementary Fig. 1) most likely result from the strongly reduced contribution of soil organic matter. Additionally, very low PAH concentrations in this interval, close or below the detection limit, might result in larger analytical uncertainties and potential mathematical artifacts. Therefore, the evaluation of PAH^7^/_5_ is more informative for events 1–8, which are characterized by high concentrations of biomarkers of both combustion and soil organic matter input; consequently, our interpretation of this ratio focuses on its mean values for these eight events (Figs. 2, 4).

Based on the above-mentioned relationships between C_2_-DBF and PAHs, the increase in PAH^7^/_5_ towards the EPME, and especially in events 7 and 8, tentatively indicates an increase in the proportion of volcanogenic PAHs relative to soil/wildfire-derived PAHs. Higher PAH^7^/_5_ values at the top of the sequence also correspond to the increased Hg/TOC as a result of the accumulation of Hg released globally by volcanic activity (mercury enrichment interval 4)^14^ on land, which was transported to the ocean upon the collapse of terrestrial ecosystems^13,18^. While volcanism might have become more intense towards the end of the sequence, we argue that the increase in PAH^7^/_5_, as well as low abundances of C_2_-DBF and prominent peaks in Al, might also be indicative of the collapse and exhaustion of the terrestrial ecosystems in response to a combination of stressors, such as acid rain, heat, drought, and wildfires^1^.

### Centennial-scale sequence of environmental deterioration

To further evaluate this hypothesis, we concentrate on the uppermost 4 cm (ca. 2000 yr) of our record, which includes the onset of the EPME as defined by the extinction of Paleozoic corals and fusulinids^32^. Statistical analysis based on eighteen fossiliferous sections in south China and peri-Gondwana (Tibet, Kashmir, and the Salt Range of Pakistan) indicates that the EPME starts from the top of Bed 24e with the main phase being Bed 25 at the GSSP Meishan section^37^. Geochronological and paleontological studies of the Penglaitan section, which is stratigraphically expanded compared to the GSSP Meishan section, further support the main phase of extinction being located at Bed 25^38^. In addition to the molecular and elemental markers described before, we include sulfur (S) abundance (also compared to iron (Fe) to identify pyrite layers) as well as existing framboidal pyrite data^39^ as indicators for euxinic conditions in the water column and/or the depositional environment^40^ (Fig. 4).

Event 4 at Bed 24e, which immediately preceded the onset of the EPME is characterized by the largest peak of soil organic matter input as indicated by C_2_-DBF; this pulse of soil organic matter input was probably associated with episodes of mass wasting concurrent with, and promoted by, wildfires. Notably, this event is distinguished by the highest values of βα/(βα + αβ) ratios for C_30_ hopane isomers, indicative of oxic-soil organic matter input^41^. The occurrence of this event is in line with the terrestrial ecosystem oxidation event, as envisaged by the Hg isotopes and modeling of Hg cycling^18^ (Fig. 2). This peak in soil organic matter is followed by a peak in clastic material, indicating that after the topsoil had been stripped, the exposed bedrock may have become more susceptible to weathering and transport to the ocean. The intensity of the C_2_-DBF signal becomes weaker towards event 8 at the top of Bed 24e, following the maximum during event 4, while PAH intensity peaks are about equal during events 4–8 (Fig. 4). After event 4, soil organic matter input thus becomes less prominent. During event 7, a smaller C_2_-DBF maximum coincides with the maximum in Al, indicating that the weathering of soil and bedrock occurred almost simultaneously, probably due to the reduced thickness of the soil soon after the major mass wasting associated with event 4. Event 4 is thus identified as a turning point of terrestrial and marine ecosystem deterioration. Events 4 and 7 finally resulted in euxinia as indicated by the accumulation of framboidal pyrite, S, Fe, and pyrite laminae. Euxinia was probably caused by fertilization through terrestrial runoff that provided excess nutrients to the marine realm and reduced dissolved oxygen due to the aerobic decomposition of planktonic organic matter. Consequently, events 4 and 7 show highly characteristic maxima in wildfires and soil weathering, closely followed by maxima in bedrock weathering and/or soil leaching, and finally, euxinia. For event 8, for which maxima in PAH abundances and PAH^7^/_5_ were recorded, Al, S, and Fe peaks are not observed, but are expected to be part of the subsequent ash bed (Bed 25-1) as pyrite laminae have been reported at the base of this bed^42^.

Our dataset records a temporal sequence of high-resolution environmental deterioration that is consistent with previous, less detailed observations and thus clearly extends the insights into the centennial to millennial-scale interplay of environmental stressors leading to the EPME. Wildfires can severely damage vegetation cover^43^, although ecosystems generally recover from these episodes relatively rapidly, i.e., within decades^1^. However, as the intensity and/or frequency of wildfires increased and was amplified by additional stressors derived from Siberian Traps volcanism (e.g., acid rain, changes in solar radiation, aridity resulting from global warming)^1,44^, they seem to have contributed to the devastation of the terrestrial ecosystem^33^. At the end of the Permian, enhanced nutrient supply to the oceans from soil runoff^17^ as a consequence of wildfires^33^ would have been combined with global warming^44^ and continental shelf weathering^45^ related to exposure of vast continental shelves due to rapid marine regression^46^. These nutrients would have led to a substantial spike in marine primary productivity and oxygen consumption, ultimately resulting in euxinic water column conditions^4,47,48^ that triggered the end-Permian mass extinction of marine metazoans. This sequence of volcanism-weathering-anoxia has also been suggested by a geochemical modeling study on much longer time scales^49^. However, we show that the interactions of environmental changes on land with those in the ocean took place at decadal to centennial time scales, and that irreversible tipping points were crossed as a consequence. Thus, the thermal evolution of the Earth’s interior taking place on long geological time scales and expressed as the Siberian Traps episode, ultimately led to abrupt environmental changes at the Earth’s surface.

### Timing of terrestrial and marine crises

The timing of the marine and terrestrial crises at the end of the Permian is under debate. In high-latitude Australian terrestrial sections, where the extinction is well studied on a multiple-basin scale, extinction occurred from 252.54 ± 0.08 to 252.10 ± 0.06 Ma^50^. However, the temporal relationship between the terrestrial and marine crises in Australia is unknown because the marine extinction cannot be well defined in the Australian predominantly continental sections. Meanwhile, numerous sections in South China (including the GSSP Meishan section) are set in a near-shore setting, thereby affording information from both the marine and terrestrial realms. At the low-latitude South China sections, the marine extinction, starting at 251.941 ± 0.037 Ma^8^, was preceded by a terrestrial crisis^51,52^. Our high-resolution analysis confirms these previous findings^51,52^ and further demonstrates how environmental stressors leading to extinctions on land and in the ocean are interrelated at the centennial to millennial scales. Namely, this study shows that the terrestrial crisis occurs ~300 yr (120**–**480 yr and ± 2 s.d.) prior to the onset of the marine extinction in South China. Thus, the terrestrial environmental degradation and extinction at high southern latitudes was 200**–**600 kyr earlier than at low latitudes. Factors that explain regional differences in the timing of extinction are unknown, but in addition to the Siberian Traps volcanism, attention is beginning to focus on the localized ecological impact of the extensive circum-Pangean felsic volcanism ‘Pangean ring of fire’ that encircled the amalgamated continents during the late Permian^48,53,54^. Further paleontological, geochemical, and chronological studies are needed to understand the regional interplay and timing of terrestrial and marine extinctions.

## Methods

### Samples

Sedimentary rock samples were taken from an outcrop of the uppermost Permian Changxing Fm. in the Meishan C section, Zhejiang Province of South China (31°4′36.74″N, 119°41′52.80″E) (Fig. 1). The depositional setting at the Meishan section was mid-slope^27^. Lithologically, the upper Permian, consisting of limestone with frequent lamination, is distinctive from the overlying Lower Triassic strata^27^ of argillaceous, soft sedimentary rocks. A diamond saw with a beforehand baked blade was used to cut the rock into subsections with minimized contamination. The size of subsections was maintained at less than 5 (height) × 15 (width) × 50 (length) mm in order to fit into the custom-made MSI sample holders. Subsections were fixed in the MSI sample holders with double-sided conducting tape and stored in a desiccator until elemental mapping and measurement by MSI. In total, we investigated a section of 20 cm in length originating from Bed 24d to Bed 25. Following previous studies^21,32^, the top of Bed 24e is set to 0 years (or 0 m).

### Age model

The Meishan section is well-constrained by high-resolution U-Pb zircon dating^8,55^. A linear high-resolution age model was constructed using a weighted-mean sedimentation rate calculated in a Monte Carlo simulation in ref. ^8^. With this mean-weighted rate of 2.6 cm/kyr (minimum: 1.6 cm/kyr; maximum: 6.5 cm/kyr) in Beds 22 to 25 in the Meishan section^8^, the resolution of our analysis corresponds to 3.9 yr (minimum: 1.5 yr; maximum: 6.3 yr), and is therefore sufficient to reveal short-term, pulsed volcanism and related environmental disturbances.

However, we note that some uncertainty remains when assuming a linear sedimentation rate since major environmental and biotic changes are recorded by the lithological changes towards the top of Bed 24. While the decrease in carbonate-producing biota towards the top of Bed 24^42^ would result in a decrease in sedimentation rate, this decrease in sedimentation rate was likely compensated by the simultaneously increased rate of soil influx and bedrock weathering^56^. The Bayesian Bchron age model for the Meishan section indicates that the sedimentation rate became progressively higher towards the top of Bed 24^57^. This would imply an increasing temporal resolution toward the top of Bed 24.

### Ultra-high-resolution mass spectrometry imaging (MSI)

A 7 T solariX XR FT-ICR-MS equipped with a Smartbeam II laser (Bruker Daltonik, Bremen, Germany) was used for MSI. The laser scanned the sample in a defined area with a distance of 100 µm between spots. Laser power, frequency, and the number of shots per spot were adjusted for optimal signal intensities.

PAHs and C_2_-DBF were identified based on their monoisotopic mass. External mass calibration was done using a NaTFA solution^58^ in positive-ion mode with electrospray ionization in a mass-to-charge range of 150-750 *m/z*, followed by an internal lock mass calibration using the *m*/*z* value 179.9995 (C_15_) in DataAnalysis 4.4 SR1 (Bruker Daltonik). Clusters composed solely of carbon atoms up to C_60_ were detected in abundance in our samples. After calibration, *m*/*z*, intensity, and signal-to-noise ratio of the targeted PAH and C_2_-DBF species were exported together with their xy-coordinates for further processing in MATLAB R2015b using a DataAnalysis Script File. To avoid storing low-intensity noise signals and assure high data quality, all MSI data were acquired with 25% data reduction. Additionally, a signal-to-noise ratio of 3 was applied as a filter. Analogous procedures for ensuring high-quality signals have been successfully used in previous studies of MSI^59,60^. Lowess smoothing of MSI data was conducted using Origin (2018) software with a span value = 0.01.

### Elemental mapping

Elemental mapping was performed on an M4 Tornado Micro-XRF spectrometer system (Bruker Nano Analytics) equipped with a micro-focused Rh source (50 kV and 600 µA) with poly-capillary optics (25-µm spot size). Measurements were conducted under vacuum (20 mb) conditions with a resolution of 50 µm per pixel and a scan time of 60 ms per pixel. Map data were initially processed and visualized with M4 Tornado Software version 1.3. Distribution maps of Al, Fe, and S (counts) were exported as xy-matrices to csv files and further processed.

### Conventional GC-MS analysis

Seven sediment samples (0 to −1 cm, −1 to −2 cm, −2 to −4 cm, −4 to −8 cm, −8 to −13 cm, −13 to −16 cm, and −16 to −20 cm) were ground with an agate mortar. The ~3 g of powdered samples were then ultrasonically extracted five times with toluene:MeOH (9:1). The extracts were transferred into 60 ml vials and carefully concentrated under a stream of N_2_. The concentrated extracts were separated into non-polar and polar fractions using silica gel chromatography. The non-polar fraction and polar fraction were obtained by elution with *n*-hexane:DCM (4:1) and DCM:MeOH (4:1), respectively. The non-polar fraction was analyzed by GC-MS on an Agilent gas chromatograph (GC, 6890) interfaced with an Agilent 5975 mass selective detection (MSD) instrument. A DB-5MS column (60 m × 250 μm × 0.25 μm) was installed with the GC oven. We used a temperature program of: isothermally at 40 °C for 2 min, ramped to 320 °C at a rate of 4°/min, and then held at this temperature for 22 min. PAHs (four-ringed PAH: pyrene and fluoranthene; five-ringed PAH: benzo[e]pyrene, benzo[a]pyrene, and benzo[b/j/k]fluoranthene; 6-ringed PAH: indeno[1,2,3-cd]pyrene and benzo[ghi]perylene; seven-ringed PAH: coronene) and C_2_-DBF were identified (Supplementary Fig. 4) based on a comparison of mass spectra and relative GC scan numbers with published data^61,62^.

### Data analysis

In order to assess the strength of periodicities of combustion events at different times and for different periods, wavelet analysis using the Morlet wavelet was performed on PAH data using the Paleontological Statics (PAST) software package developed by ref. ^63^. The Pearson’s correlation test was used to explore collinearity between biomarkers and *p* values were calculated to evaluate statistical significance.

## Supplementary information

Supplementary Information

## Data Availability

All data generated in this study have been deposited in the PANGAEA data repository under access code 10.1594/PANGAEA.952297.

## Code availability

The latest version of the PAST software package is available at https://www.nhm.uio.no/english/research/resources/past/ along with the User’s manual to generate the results for the paper. The code in the Past is based on Torrence and Compo^64^.
