# Supplementary material for: Centennial scale sequences of environmental deterioration preceded the end-Permian mass extinction
Source: Nat Commun. 2023 Apr 14;14:2113. doi: 10.1038/s41467-023-37717-0 (PMC10104797; doi:10.1038/s41467-023-37717-0)
Supplement: Supplementary file 1 — Supplementary Information [file 41467_2023_37717_MOESM1_ESM.pdf]

Supplementary Information

"Centennial scale sequences of environmental deterioration preceded the end-Permian mass extinction"

Ryosuke Saito[1,2,3,4], Lars Wörmer[1], Heidi Taubner[1], Kunio Kaiho[5], Satoshi Takahashi[6], Li Tian[7], Masayuki Ikeda[8], Roger E. Summons[2], Kai-Uwe Hinrichs[1]

[1]MARUM - Center for Marine Environmental Sciences & Faculty of Geosciences, University of Bremen, 28359 Bremen, Germany

[2]Department of Earth, Atmospheric and Planetary Sciences, Massachusetts Institute of Technology, 45 Carleton Street, Cambridge, MA 02142, USA

[3]Department of Geosphere Sciences, Yamaguchi University, 1677-1 Yoshida, Yamaguchi City, 753-8512, Japan

[4]Japan Science and Technology Agency, PRESTO, 4-1-8 Honcho, Kawaguchi, Saitama 332-0012, Japan

[5]Department of Earth Science, Tohoku University, Sendai 980-8578, Japan

[6]Department of Earth and Environmental Sciences, Graduate School of Environmental Studies, Nagoya University 464-8601, Japan

[7]The Key Laboratory of Biogeology and Environmental Geology and Faculty of Earth Science, China University of Geosciences Wuhan 430074, China

[8]Department of Earth and Planetary Science, University of Tokyo, Bunkyo, 113-0033, Japan

Supplementary Figures

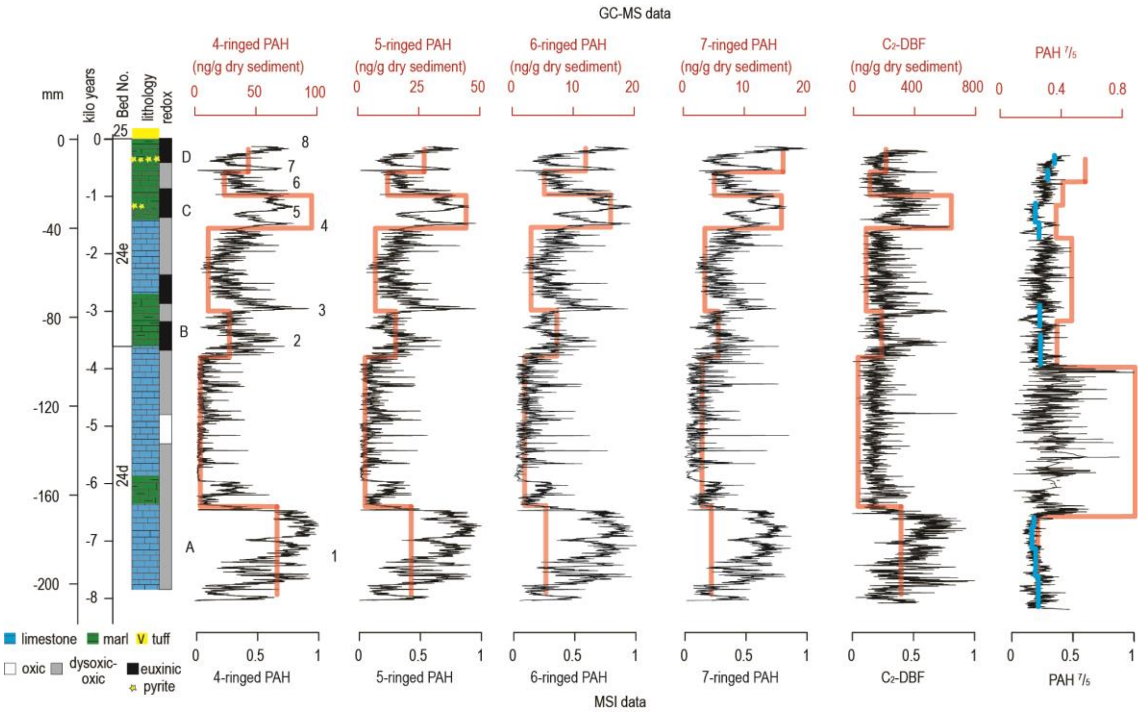

Supplementary Fig. 1. Stratigraphic variations of polyaromatic hydrocarbons (PAHs) and the PAH $^{7}/_{5}$ ratio. The 4- to 7-ringed PAHs and PAH $^{7}/_{5}$ are shown as markers for combustion and combustion temperature, respectively. Given the sensitivity of PAH $^{7}/_{5}$ to intervals with low contribution of soil-derived organic matter, as well as the increased analytical uncertainty and risk of mathematical artifacts when concentration of PAHs is low, Figs. 2 and 4 only show PAH $^{7}/_{5}$ values for the eight major combustion events characterized by increased PAH concentrations. Thereby changes in the nature of these events towards the end-Permian mass extinction can be assessed. See Results and Discussion in text for details. Note that the offset between GC-MS and MSI data of PAH $^{7}/_{5}$ are most probably related to the averaging of signals inherent to conventional analysis. This averaging is biased towards stratigraphic levels in which concentrations of the targeted compounds are higher[1]. PAHs for MSI-based data are normalized to their maximum values. The vertical red lines on each plot are the corresponding depth range averaged data of GC-MS data for each sample, black lines show MSI-based data with 0.1 mm resolution. The vertical blue lines on PAH $^{7}/_{5}$ show the mean values of the PAH $^{7}/_{5}$ of MSI-based data for each event. Letters A-D identify intervals of increased PAH concentrations, while numbers 1-8 identify single events of increased PAH input. The redox bar is after framboidal pyrite data[2].

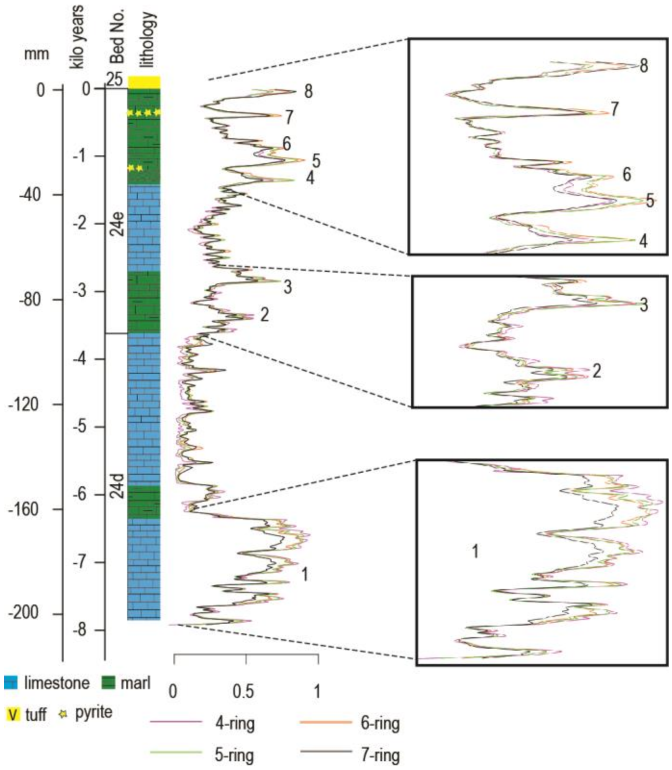

Supplementary Fig. 2. Superposition of changes in stratigraphic variations of PAHs by MSI-based data. The 4- to 7-ringed PAHs are shown as combustion markers. Notice that 7-ringed PAH is the highest value in event 8.

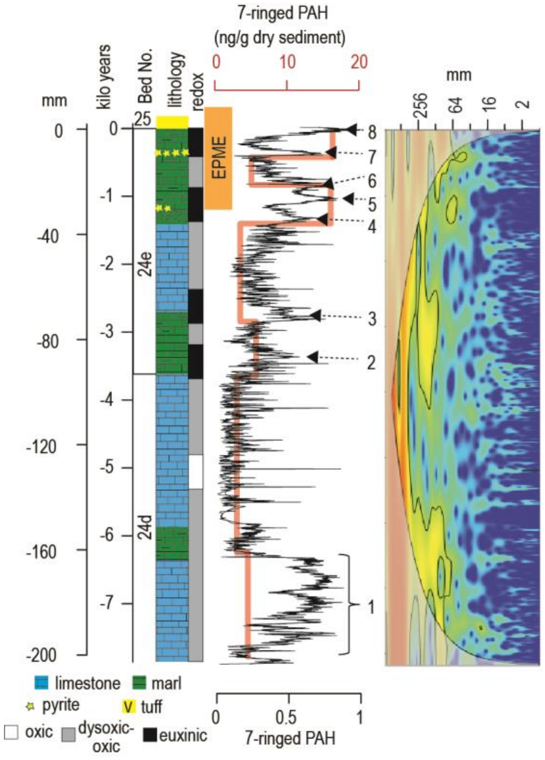

Supplementary Fig. 3. Stratigraphic variations of 7-ringed PAH with wavelet analysis. The 7-ringed PAH values are shown as combustion markers. Statistically significant frequencies in the range of ca. 32 to 256 mm corresponding to the centennial-scale (c. 400~2000 yr) are identified for events 1–8, with higher frequencies towards the end-Permian mass extinction (EPME). A linear sedimentation rate[2] is applied. Note, the actual frequency of combustion detected in the upper 40 mm could be higher, since the sedimentation rate in the upper sequence could be higher than in the lower (see Method section in text). The PAH values for MSI-based data are normalized to their maximum values. The red line is data from conventional GC-MS while the black line shows MSI-based data with 0.1 mm resolution. Spectral power is shown in blue (weak) to red (strong). The black contour for wavelet spectrum is the 90% confidence level. The black curved line represents a cone of influence, in which the information outside (the pale area) is affected by boundary effects[3]. The wavelet analysis is carried out using software PAleontological Statistics (PAST) developed by Hammer et al.[3].

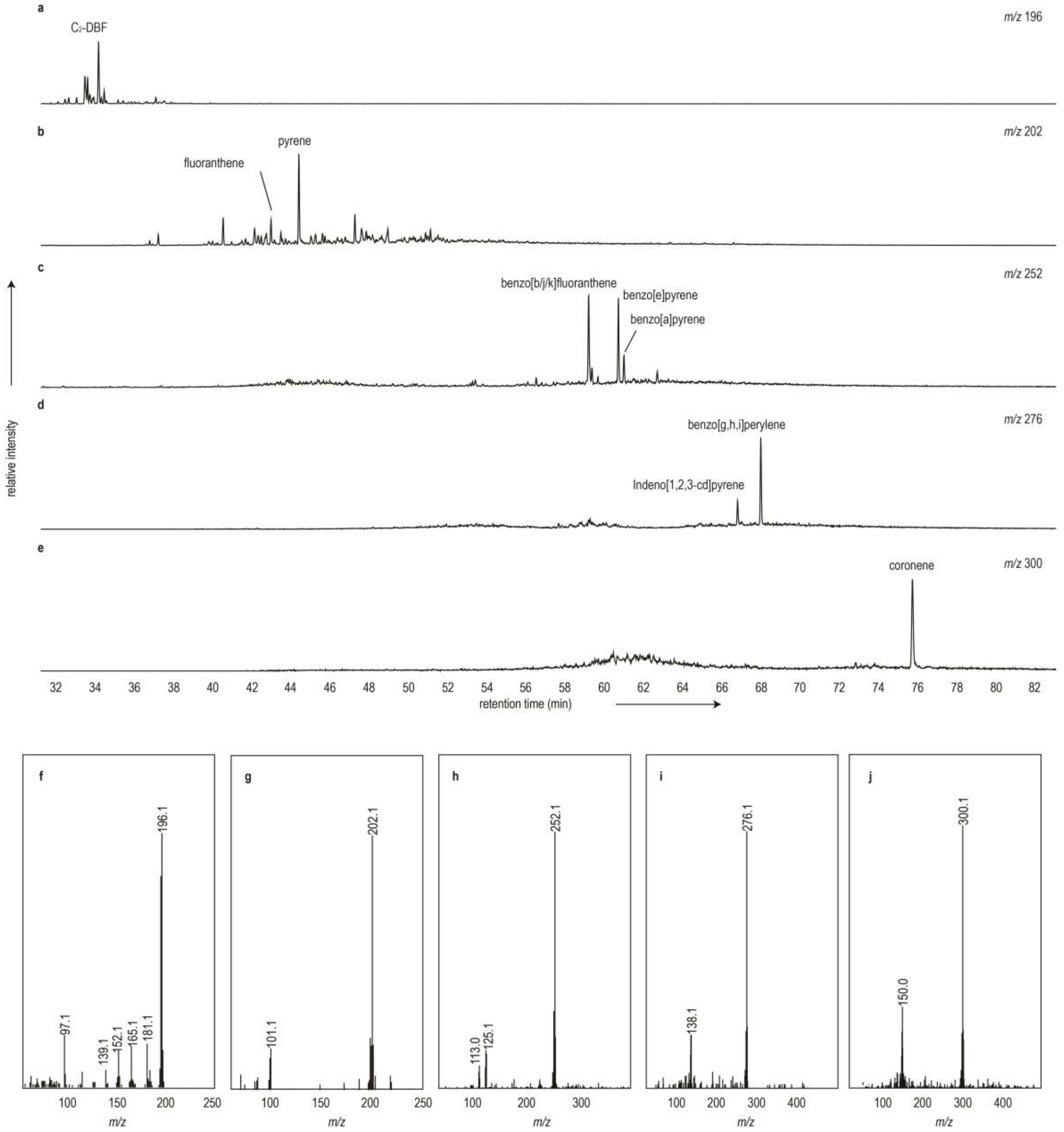

Supplementary Fig. 4. Representative mass chromatograms and spectra from the GC-MS analysis of the Meishan section samples: **a**-**e**, mass chromatograms of C_2-DBF (*m/z* 196) (**a**), 4-ringed PAHs (*m/z* 202) (**b**), 5-ringed PAHs (*m/z* 252) (**c**), 6-ringed PAHs (*m/z* 276) (**d**), 7-ringed PAH (*m/z* 300) (**e**). **f**-**j**, mass spectra of C_2-DBF (**f**), pyrene (**g**), benzo[e]pyrene (**h**), benzo[ghi]perylene (**i**), and coronene (**j**).

Supplementary Tables

Supplementary Table 1. Duration of combustion events. A plausible range for the duration of individual combustion events was estimated using weighted-mean (2.6 cm/kyr), minimum (1.6 cm/kyr) and maximum (6.5 cm/kyr) sedimentation rates reported in Burgess et al.[4].

Event number	Duration (mean) (yr)	Duration (minimum) (yr)	Duration (maximum) (yr)
8	140	51	230
7	170	49	270
6	110	45	180
5	210	85	370
4	260	100	420
3	370	140	600
2	510	200	820
1e	160	65	260
1d	370	140	600
1c	340	130	550
1b	92	32	150
1a	530	200	860
mean	270	-	-

Supplementary Discussion

This discussion reviews the origin of PAHs peaks observed in Bed 24 at the Meishan section. There are three possibilities for the origin of the PAHs peaks: redeposition, slow sedimentation rates, or a direct indication of forest fires that occurred at the time of deposition.

First of all, the depositional setting of the Meishan section is a carbonate ramp slope[5], where the redeposited materials are derived from a shallow carbonate platform that produces shallow-marine carbonates (purer carbonates) rather than siliciclastic materials. Even if siliciclastic materials were transported into the carbonate ramp slope from the shallow-marine carbonate platform, they do not necessarily have to be rich in PAHs and C_2-DBF. Additionally, oxidative redeposition of carbonate rocks occurs during redeposition due to the introduction of oxygen, a process referred to as the "burn-down effect”[6-8]. In fact, sedimentological studies have reported that redeposits at the Meishan section are deposited as such oxidative deposits[8]. Thus, terrestrial source materials such as PAHs, C_2-DBF, and aluminum are unlikely to accumulate in redeposited sediments at the Meishan setting. Additionally, chemical tracers such as mercury[9], $\beta\alpha/(\beta\alpha + \alpha\beta)$ ratios for C_{30} hopane[10] and $\delta^{13}C_{carb}$[11] exhibit a stratigraphic distribution with peaks only in the uppermost part of Bed 24, which clearly cannot have been introduced by redeposition from lower stratigraphic levels.

The remaining possibilities are the slow sedimentation rate or the indication of forest fires that occurred at the time of deposition. Based on the time course of the forest fire event, we believe the latter is more likely. In general, the rate of soil runoff experiences a surge immediately after a forest fire[12]. Given that the enrichment of PAHs coincided with terrestrial soil-organic matter (C_2-DBF) and aluminum, it is more plausible to assume that PAHs were deposited by an augmented supply from terrestrial sources, caused by a short-lived surge in soil runoff following the forest fires, rather than through very slow deposition. Notably, since events 4-8 were deposited during a period of amplified sedimentation rates (see: Age model in Methods), it is more probable that they were deposited rapidly, rather than being concentrated as a result of a slow deposition process. In addition, evidence of enhanced continental weathering rates and soil inflow as a consequence of the EPME[13] also contrasts with very slow sedimentation rates.

Supplementary References

1 Alfken, S. *et al.* Mechanistic Insights Into Molecular Proxies Through Comparison of Subannually Resolved Sedimentary Records With Instrumental Water Column Data in the Santa Barbara Basin, Southern California. *Paleoceanography and Paleoclimatology* **35**, doi:10.1029/2020pa004076 (2020).

2 Wei, H., Zhang, X. & Qiu, Z. Millennial-scale ocean redox and δ13C changes across the Permian–Triassic transition at Meishan and implications for the biocrisis. *International Journal of Earth Sciences* **109**, 1753-1766, doi:10.1007/s00531-020-01869-x (2020).

3 Hammer, Ø., Harper, D. A. T. & Ryan, P. D. PAST-palaeontological statistics, ver. 1.89. *Palaeontologia Electronica* **4**, 1-9 (2001).

4 Burgess, S. D., Bowring, S. & Shen, S.-Z. High-precision timeline for Earth's most severe extinction. *Proceedings of the National Academy of Sciences* **111**, 3316-3321, doi:10.1073/pnas.1317692111 (2014).

5 Chen, Z.-Q., Tong, J., Liao, Z.-T. & Chen, J. Structural changes of marine communities over the Permian–Triassic transition: Ecologically assessing the end-Permian mass extinction and its aftermath. *Global and Planetary Change* **73**, 123-140, doi:10.1016/j.gloplacha.2010.03.011 (2010).

6 Keil, R. G., Hu, F. S., Tsamakis, E. C. & Hedges, J. I. Pollen in marine sediments as an indicator of oxidation of organic matter. *Nature* **369**, 639-641, doi:10.1038/369639a0 (1994).

7 De Lange, G. J. Oxic vs. anoxic diagenetic alteration of turbiditic sediments in the Madeira Abyssal Plain, eastern North Atlantic. *Proceedings of the Ocean Drilling Program, Scientific Results* **157**, 573-579 (1998).

8 Li, G., Wang, Y., Shi, G. R., Liao, W. & Yu, L. Fluctuations of redox conditions across the Permian–Triassic boundary—New evidence from the GSSP section in Meishan of South China. *Palaeogeography, Palaeoclimatology, Palaeoecology* **448**, 48-58, doi:10.1016/j.palaeo.2015.09.050 (2016).

9 Grasby, S. E. *et al.* Isotopic signatures of mercury contamination in latest Permian oceans. *Geology* **45**, 55-58, doi:10.1130/g38487.1 (2017).

10 Cao, C. *et al.* Biogeochemical evidence for euxinic oceans and ecological disturbance presaging the end-Permian mass extinction event. *Earth and Planetary Science Letters* **281**, 188-201, doi:10.1016/j.epsl.2009.02.012 (2009).

11 Xie, S. *et al.* Changes in the global carbon cycle occurred as two episodes during the Permian–Triassic crisis. *Geology* **35**, doi:10.1130/g24224a.1 (2007).

12 Moody, J. A. & Martin, D. A. Initial hydrologic and geomorphic response following a wildfire in the Colorado Front Range. *Earth Surface Processes and Landforms* **26**, 1049-1070, doi:10.1002/esp.253 (2001).

13 Algeo, T. J. & Twitchett, R. J. Anomalous Early Triassic sediment fluxes due to elevated weathering rates and their biological consequences. *Geology* **38**, 1023-1026, doi:10.1130/g31203.1 (2010).
